# Insight into de-regulation of amino acid feedback inhibition: a focus on structure analysis method

**DOI:** 10.1186/s12934-023-02178-z

**Published:** 2023-08-23

**Authors:** Sadia Naz, Pi Liu, Umar Farooq, Hongwu Ma

**Affiliations:** 1 Biodesign Center, Tianjin Institute of Industrial Biotechnology, Chinese Academy of Sciences, Tianjin, 300308 China; 2 Department of Chemistry, COMSATS University Islamabad, Abbottabad Campus, Islamabad, 22060 Pakistan

**Keywords:** Amino acid biosynthesis, Feedback inhibition, In vitro mutagenesis, De-regulation, Protein structure analysis

## Abstract

Regulation of amino acid’s biosynthetic pathway is of significant importance to maintain homeostasis and cell functions. Amino acids regulate their biosynthetic pathway by end-product feedback inhibition of enzymes catalyzing committed steps of a pathway. Discovery of new feedback resistant enzyme variants to enhance industrial production of amino acids is a key objective in industrial biotechnology. Deregulation of feedback inhibition has been achieved for various enzymes using in vitro and in silico mutagenesis techniques. As enzyme’s function, its substrate binding capacity, catalysis activity, regulation and stability are dependent on its structural characteristics, here, we provide detailed structural analysis of all feedback sensitive enzyme targets in amino acid biosynthetic pathways. Current review summarizes information regarding structural characteristics of various enzyme targets and effect of mutations on their structures and functions especially in terms of deregulation of feedback inhibition. Furthermore, applicability of various experimental as well as computational mutagenesis techniques to accomplish feedback resistance has also been discussed in detail to have an insight into various aspects of research work reported in this particular field of study.

## Introduction

Product feedback inhibition of allosteric enzymes is of paramount importance in biotechnological industries for discovery of efficient microbial strains for increased production of metabolites of interest. Discovery of product-feedback inhibition traced back to 1950’s and was first reported by Novick and Szilard for the tryptophan biosynthetic pathway and has been reported as cornerstone for regulation of cell functions and control fluxes for optimal growth in microorganisms [1, 2]. Allosteric regulation of proteins is a fundamental mechanism of cellular control e.g. regulation of the enzymes involved in biosynthesis of amino acids, nucleotides and vitamins. In case of amino acid feedback inhibition, the first enzyme in the pathway is an allosteric enzyme that binds to the end product (i.e. amino acid) which alters its active site so that it cannot mediate the enzymatic reaction needed to initiate the pathway. Ultimately, pathway is shut down as long as adequate amounts of the end product are present but inhibition is relieved and the enzyme regains its activity if the end product is used up or disappears. Owing to role of amino acids as building block of life and their wide applications in agriculture, pharmaceutical and cosmetics industries, the chemical industry is focused on various synthetic strategies for production of these biochemically distant compounds. Amino acids production industry is growing day by day at an annual rate of 7%, as reported previously [3]. Allosteric feedback inhibition of the committed step in amino acids biosynthetic pathways is thought to maintain homeostasis of end-products [4]. The consequences of dysregulating these enzymes were mainly studied in vitro [5] or in the context of biotechnological overproduction strains [6].

Extensive studies have been reported for deregulation of feedback inhibition of various amino acids biosynthetic pathways using structural information of target enzyme, in vitro and in silico mutagenesis techniques. Most allosterically regulated enzymes are oligomeric in structural composition (i.e. made up of two or more polypeptide chains) having more than one active site and allosteric sites. Although reviews over the metabolic engineering strategies of amino acid biosynthetic pathways have been reported previously [7] still structural and functional aspects of feedback inhibition is not reviewed yet. An enzyme’s function is intrinsically linked to its three dimensional structure, determining how it performs substrate binding, catalysis and regulation. The relation of enzyme structure to its functions signifies the importance of structural insights of enzyme. Keeping in view the importance of structural aspects of enzyme, here, we provide detailed insight into various amino acid biosynthetic pathways, structural details of target enzymes feedback inhibited by amino acid (each case), mutagenesis approaches (both in vitro and in silico) used to incorporate structural and conformational changes to deregulate their inhibition tendency. The structure, design and mechanism of product feedback inhibition for all enzyme targets have been discussed in detail to provide an insight into structural and functional basis of amino acid feedback inhibition. Current review will provide guidelines for designing of better feedback resistant enzymes and will facilitate biotechnologists for discovery of novel enzyme variants for increased production of amino acids at industrial scale.

## Small molecule regulation of enzyme activities

Regulation of enzyme activity has profound effects on cell functions with huge practical applications. Enzyme activities can be regulated either by dissociation or binding of regulators and effectors causing conformational or structural changes that ultimately determine their catalytic activity [8, 9]. Small molecule regulation of enzyme functions has garnered much interest and plenty of new molecules have been identified with specific regulatory functions. Use of small molecule for controlling gene-product activity to precisely elucidate functions of protein target has huge significance and is termed as chemical genetics. Protein functions are regulated in two ways (1) loss of function using small molecule inhibitors, (2) gain of function using small molecule activators [10]. Cell metabolisms are controlled by various metabolic pathways and interlinked networks to generate energy alongside biomass that is transmitted to other neighboring cells. Two well established methods to regulate cell metabolism are: (a) genetic regulation, (b) small molecule inhibition or activation (For instance allosteric inhibition) [11–13].

Allosteric regulation controls given protein activity involved in catalysis, signal transduction, gene regulation alongside various other biological processes [14, 15]. Regulation of protein functions or dynamics due to binding of regulator at site other than enzyme’s active site is termed as “allostery”. Allosteric regulators are classified as allosteric activators and allosteric inhibitors leading to increase in protein’s activity and decrease in activity, respectively. Allosteric proteins have capacity to switch between two states i.e. active state and inactive state triggered by allosteric signal (an effector/binder) [16]. Control of protein activity by these allosteric effectors is attributed to their ability to stabilize specific conformation of target protein with distinct binding. Most protein surfaces have various potential allosteric sites except fibrous as well as structural proteins [14]. For instance, the binding of oxygen at one part of hemoglobin increases binding tendency of oxygen at other subunit represents most suitable example of allostery [17]. Although most of allosteric inhibitors have been discovered serendipitously but have more selectivity as compared to orthosteric ones [18–20].

**Fig. 1:**
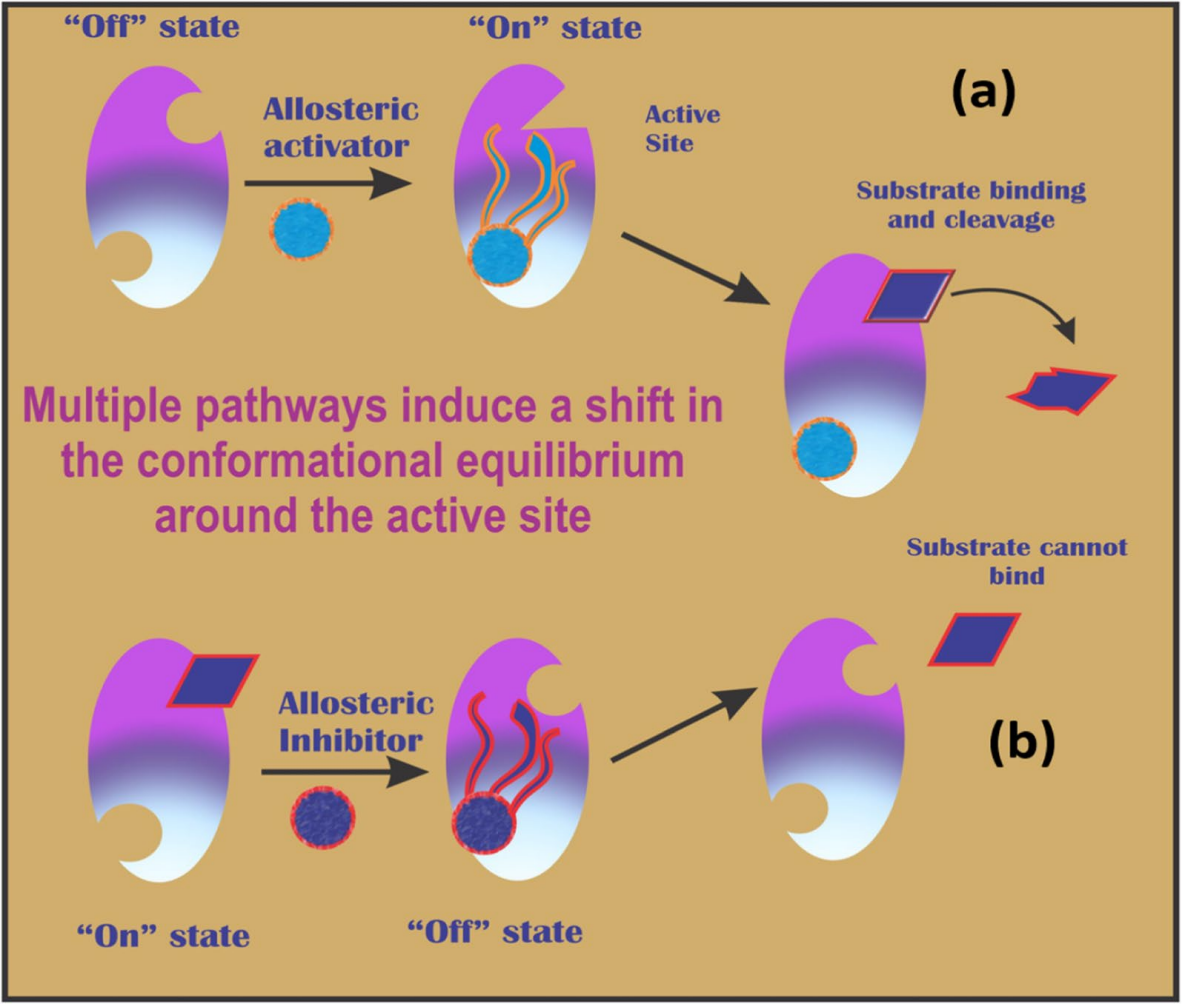
Mechanism of Allosteric Regulation in proteins **a**. Allosteric activator induces conformation changes to facilitate in substrate binding, **b**. Allosteric inhibitor reduces substrate binding tendency through conformation changes

Initially, the allosteric property was reported in quaternary proteins but later it has been confirmed as intrinsic characteristic of all dynamic proteins. Keeping in view the existence of these proteins as collection of active and inactive conformers, the binding of allosteric regulators causes structural changes in proteins and shifts this dynamic equilibrium either towards an active state (allosteric activator) or inactive state (allosteric inhibitor) [14, 21] as depicted in Fig. 1. Although allosteric inhibition being advantageous over competitive inhibition is more desirable but their mechanism of action is not clearly defined. Role of allosteric proteins as intermediate for signal transduction pathways is well documented where they serve as mediator, modulator or adaptor to activate partner proteins to perform their activity [22].

## Amino acid biosynthetic pathway

Amino acids are building blocks of proteins and are critical for life as they play role in synthesis of various metabolites through metabolic pathways. Amino acids biosynthesis is a complex array of alternate pathways connected as non-linear series of reactions [23]. Apart from being precursor for protein synthesis, amino acids also serve as intermediates of other biosynthetic pathways of cell like purine synthesis [24, 25]. In plants, they are catabolized through tricarboxylic acid pathway to produce energy that is utilized by cells for their growth and proliferation [26, 27].

The biosynthetic pathways for the essential amino acids (i.e. acquired through dietary sources; animals cannot synthesize) are found only in microorganisms and are more complex as compared to non-essential amino acids. Owing to their common metabolic precursors, the amino acids have been classified as four families namely Aspartate family (lysine, methionine, threonine, Asparagine) [28–30], Pyruvate family (Alanine, leucine, isoleucine, valine) [31], Aromatic family (phenylalanine, Tyrosine, Tryptophan) [32, 33] and α-ketoglutarate family (glutamic acid, glutamine, proline, arginine) along with Histidine and serine [34].

Availability of given amino acids in living organisms are effected by either regulatory factors being capable of controlling synthesis of amino acids or either their proficient catabolism [35]. So far, over 300 amino acids have been reported out of which 20 amino acids serve as basic structural units of proteins while 10 amino acids are essential for humans and other living beings as they need to be provided through dietary sources [36, 37]. Role of amino acids in food industry, fodder, cosmetic industry, and pharmaceutical industry signifies their importance and need of high scale production to achieve market demand. Keeping in view the important role of amino acids as building block of life in human beings and animals, the chemical industry focused on various synthetic strategies for production of these biochemically distant compounds.

**Fig. 2:**
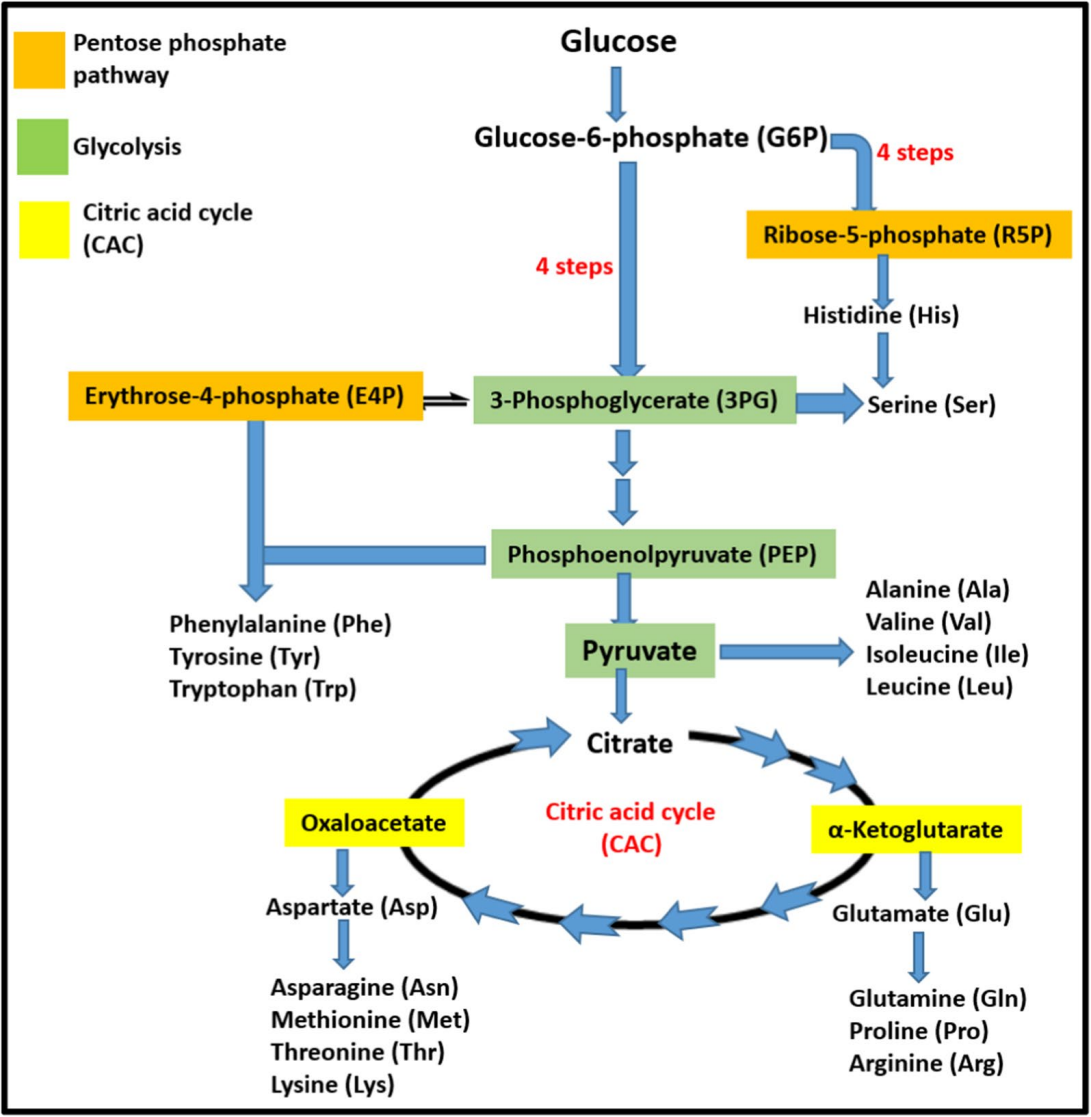
Amino acids biosynthetic pathways

Different metabolic pathways involved in synthesis of amino acids are glycolysis; for branched chain amino acids (Val, Leu) alongside Ala, Gly, Cys, Ser synthesis, citric acid cycle; for Asn, Asp, Lys, Met, Thr and Ile, phosphoribosyl pyrophosphate (PRPP) pathway; for aromatic amino acid (Phe, Trp, Tyr) and histidine synthesis and shikimate pathway [38, 39]. The Shikimate biosynthetic pathway also referred as prechorismate pathway leads to synthesis of aromatic amino acids (AAA) having huge significance in terms of their role in synthesis of various secondary metabolites (i.e. serotonin and various neurotransmitters) [40, 41]. As mentioned earlier different pathways leading to synthesis of amino acids of all families are interconnected (Fig. 2) like pyruvate an important precursor through series of enzymatic reactions is converted into alanine, valine and leucine while oxaloacetate obtained from pyruvate leads to production of aspartic acid that is ultimately transformed into asparagine, methionine, threonine, lysine and isoleucine. Similarly, phosphoenolpyruvate serve as precursor for AAA while α-ketoglutarate give rise to glutamic acid, glutamine, proline and arginine. In addition, 3-phosphoglycerate follow multistep enzyme reactions to produce phosphoserine that is later converted to serine, cysteine and glycine. The details of amino acid biosynthesis in diatoms has been reviewed previously by Mariusz A. Bromke [42]. Current review is focused in structural aspects of enzymes involved in feedback inhibition and regulation of biosynthetic pathway of amino acids.

Despite the role of amino acids for accurate functioning of cells, homeostasis is maintained through metabolic regulation of biosynthetic pathways achieved either through (i) controlled synthesis of enzyme or (ii) feedback inhibition of enzyme by end product (i.e. amino acid). This end product inhibition of enzymes is counteracted either by sufficient availability of substrate or by heavy metal cation to facilitate balanced concentration of given metabolite/intermediate/amino acid for proper functioning of cell [16, 39, 43, 44].

## Feedback inhibition of amino acids biosynthetic pathway

Discovery of product feedback inhibition dated back to 1950, has been attributed as cornerstone of metabolic regulation that has been reported to sufficiently control metabolic fluxes to facilitate proficient growth [1, 2]. For a given biosynthetic pathway, the inhibition of first committed step by end product is termed as feedback inhibition while inhibition at sites other than active sites is termed as allosteric inhibition with huge biological significance. Binding of allosteric regulator to enzyme causes conformational changes in enzyme structure and perturb its activity [35]. Allosteric inhibition of amino acids biosynthetic pathways is well documented and 16 amino acids have ability to feedback inhibit their own biosynthetic pathway by targeting allosteric sites of enzyme catalyzing first committed step. Deregulation of feedback inhibition is of utmost importance to maintain cell homeostasis and has been mostly reported in vitro studies to improve production of various amino acids with huge industrial impact [4, 45]. The details of amino acids alongside enzyme target with feedback inhibition has been summarized in Table 1.

**Table 1:** Structural information of Enzymes targeted by amino acid feedback inhibition retrieved from UniProt database

Organism	EC number	AA synthesis pathway	AA end product inhibition	Cross-reference (PDB)	3D-structure	Reference
*Mycobacterium tuberculosis*	2.3.1.1	Arginine	Arg	5YGE;5YO2;6ADD;		[46, 47]
*Thermotoga maritima*	2.7.2.8	Arginine	Arg	2BTY;		
*Saccharomyces cerevisiae*	1.2.1.38; 2.7.2.8	Arginine	Arg	3ZZF;3ZZG;3ZZH;3ZZI;4AB7;		
*Escherichia coli*	2.3.1.30	Cysteine	Cys	1T3D;		[48]
*Salmonella typhimurium*	2.3.1.30	Cysteine	Cys	4LI3;4NU8;4ZU1;4ZU6;5DBE;6AIF;		
*Methanothermobacter thermautotrophicus*	2.4.2.17	Histidine	His	2VD3;		[49–51]
*Mycobacterium tuberculosis*	2.4.2.17	Histidine	His	1NH7;1NH8;5LHT;5LHU;5U99;		
*Campylobacter jejuni*	2.4.2.17	Histidine	His	4YB5;4YB6;4YB7;5UB9;5UBG;5UBH;5UBI;		
*Escherichia coli*	2.4.2.17	Histidine	His	1H3D;1Q1K;		
*Escherichia coli*	4.3.1.19	Isoleucine	Ile	1TDJ;		[52]
*Leptospira interrogans*	2.3.1.182	Isoleucine	Ile	3BLE;3BLF;3BLI;3F6G;3F6H;		
*Escherichia coli*	2.2.1.6	Isoleucine	Val	2F1F;		[53]
*Saccharomyces cerevisiae*	2.2.1.6	Isoleucine	Val	1JSC;1N0H;1T9A;1T9B;1T9C;1T9D;5FEM;5IMS;5WKC;6BD3;6BD9;6U9D;		
*Sulfolobus acidocaldarius*	2.3.3.14	Lysine	Lys	6KTQ;	AKIII	[54]
*Thermus thermophilus*	2.3.3.14	Lysine	Lys	2ZTJ;2ZTK;2ZYF;3A9I;		
*Escherichia coli*	2.7.2.4	Lysine	Lys	2J0W;2J0X;		
*Corynebacterium glutamicum*	2.7.2.4	Lysine	Lys	2DTJ;3AAW;3AB2;3AB4;		
*Escherichia coli*	2.7.2.4; 1.1.1.3	Lysine	Thr	6MX1;	AK	[55]
*Mycobacterium tuberculosis*	2.7.2.4	Lysine	Thr	3S1T;4GO5;4GO7;		
*Thermus thermophilus*	2.7.2.4	Lysine	Thr	2DT9;2ZHO;		
*Escherichia coli*	1.2.1.11	Lysine	Cys	1BRM;1GL3;1T4B;1T4D;		[56]
*Agrobacterium fabrum*	4.3.3.7	Lysine	Lys	4I7U;4I7V;4I7W;	DHDPS	[57]
*Escherichia coli*	4.3.3.7	Lysine	Lys	1DHP;1S5T;1S5V;1S5W;1YXC;1YXD;2A6L;2A6N;2ATS;2OJP;2PUR;3C0J;3DEN;3DU0;3I7Q;3I7R;3I7S;4EOU;5T25;5T26		
*Mycobacterium tuberculosis*	4.3.3.7	Lysine	Lys	1XXX;3L21;5J5D;		
*Neisseria meningitidis serogroup B*	4.3.3.7	Lysine	Lys	3FLU;		
*Rhizobium meliloti*	4.3.3.7	Lysine	Lys	2VC6;		
*Escherichia coli*	4.4.1.13; 4.4.1.28	Methionine	Cys	1CL1;1CL2;2FQ6;2GQN;4ITG;4ITX;	CBL	[58]
*Thermus thermophilus*	2.5.1.-	Methionine	Met	2CTZ;		
*Rhodococcus sp.*	1.4.1.20	Phenylalanine	Phe	1BW9;1BXG;1C1D;1C1X;		[59]
*Escherichia coli*	5.4.99.5; 4.2.1.51	Phenylalanine	Phe	1ECM;5VHT;		
*Escherichia coli*	2.7.2.11	Proline	Pro	2J5T;2J5V;2W21;		[60]
*Escherichia coli*	1.1.1.95; 1.1.1.399	Serine	Ser	1PSD;1SC6;1YBA;2P9C;2P9E;2P9G;2PA3;		[61]
*Arabidopsis thaliana*	2.6.1.52	Serine	Cys	6CZX;6CZY;6CZZ;	PSAT1	[62]
*Mycobacterium tuberculosis*	4.1.3.27	Tryptophan	Trp	5CWA;	AS	[63]
*Mycolicibacterium smegmatis*	4.1.3.27	Tryptophan	Trp	7BVD;		
*Saccharolobus solfataricus*	4.1.3.27	Tryptophan	Trp	1QDL;		
*Salmonella typhimurium*	4.1.3.27	Tryptophan	Trp	1I1Q;		
*Serratia marcescens*	4.1.3.27	Tryptophan	Trp	1I7Q;1I7S;		
*Salmonella typhimurium*	4.1.3.27; 2.4.2.18	Tryptophan	Trp	1I1Q;		

Note: Abbreviations are used for names of enzymes with 3D-structure

Few amino acids have the tendency to feedback inhibit multiple enzyme targets and their deregulation signifies their role to improve industrial production by identifying new and better strains. For instance, arginine and proline synthesized as final product from oxaloacetate pathway feedback inhibit N-acetyl-L-glutamate kinase (NAGK) and N-acetyl-L-glutamate synthase (in case of arginine) and glutamate kinase in case of proline [64]. Similarly, lysine has capacity to feedback inhibit multiple enzyme targets namely diaminopimilate decarboxylase, dihydrodipicolinate synthase, homocitrate synthase and aspartokinase III [65, 66]. In few cases, same enzyme is targeted by multiple amino acids as feedback inhibitors like aspartokinase is feedback inhibited by both lysine and threonine [67]. Amino acid biosynthetic pathway and effect of feedback inhibition alongside use of various approaches for deregulation of inhibition has remained focus of researchers especially in industrial biotechnology. Various reports over deregulation of feedback inhibition of individual biosynthetic pathways have been previously reported by our research center. For instance, Geng et al., reported successful alleviation of feedback inhibition of DHDPS_*E.coli*_ through mutations at inhibitor binding site and identified *E. coli* MG1655 strain with improved L-lysine production yield by 46%. They utilized structural characteristics of L-lysine-sensitive DHDPS _*E.coli*_ and L-lysine-insensitive DHDPS_*C.glutamicum*_ and reported new enzyme variants through point mutations at specified sites with improved lysine production [68]. Similarly, Zhen et al., utilized combination of SCA and MD approach to accomplish successful deregulation of the allosteric inhibition of aspartokinase i.e. an industrial enzyme for increased amino acid production [69]. Recently, we have used the computational mutagenesis method to identify new mutant structures with potential deregulated feedback inhibition by tryptophan for anthranilate synthase from *S. marcescens* (Sadia et al., under revisions).

Here, we summarized structural characteristics of various enzyme targets and effect of mutations on their structures and functions especially in terms of deregulation of feedback inhibition. Applicability of various experimental as well as computational techniques (i.e. site directed mutagenesis, site specific mutagenesis etc.) to accomplish feedback resistance has also been discussed in details to have an insight into various aspects of research work reported in this particular field of study.

## Structural characteristics of enzymes targeted by amino acid feedback inhibition

**Fig. 3:**
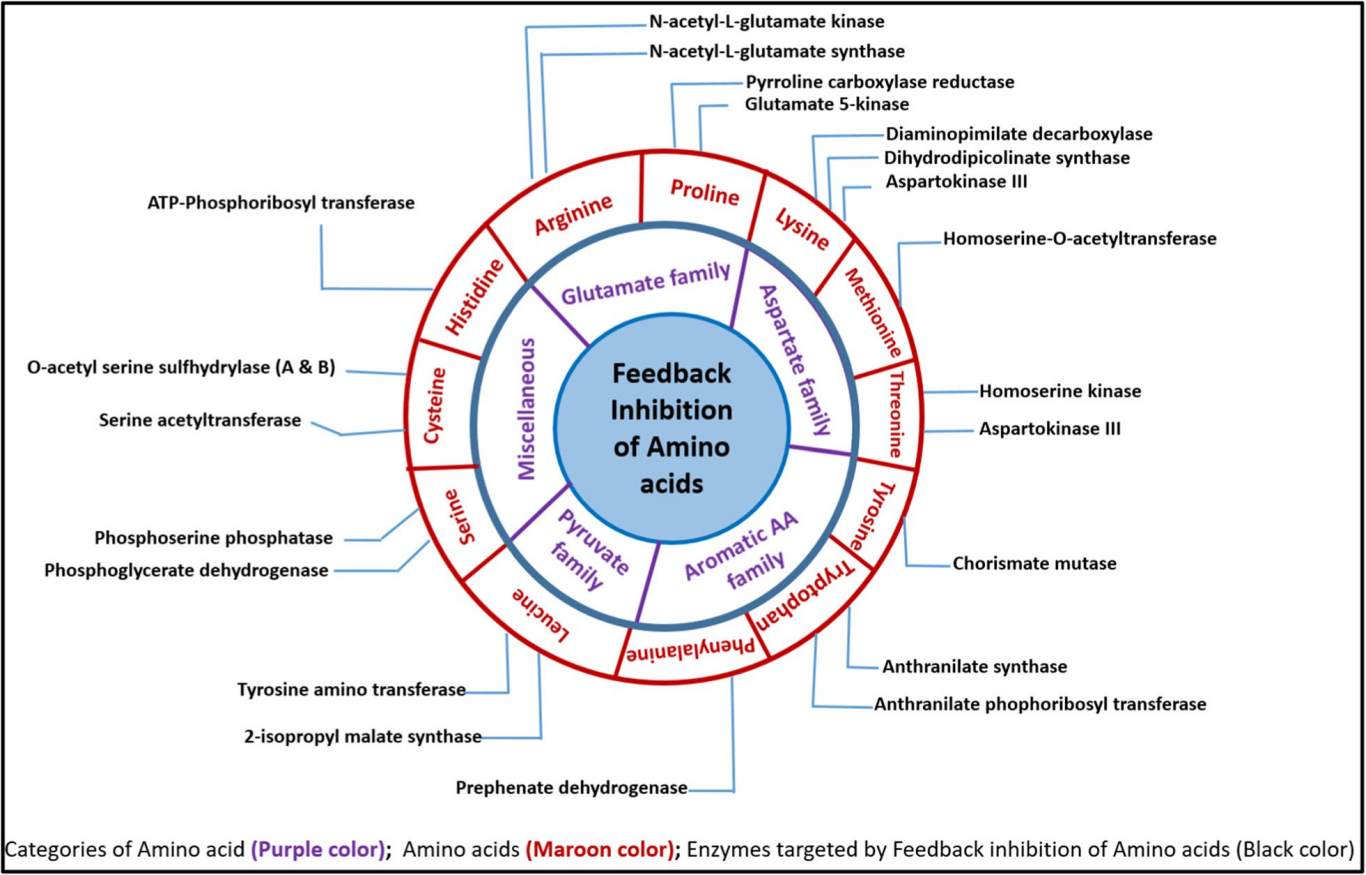
Schematic Representation of Amino acid’s Feedback Inhibition

The amino acids can be broadly grouped as four categories or families following different pathways for their synthesis. The details of amino acids capable of inhibiting committed step of their own biosynthesis, their categories, pathways as per their common precursors and their feedback sensitive enzyme targets are shown schematically in Fig. 3.

Detailed structural features of various enzymes belonging to different families of amino acids have been provided in next section.

### Glutamate family of amino acids

#### Structural insights into enzyme targeted by feedback inhibition of arginine

L-arginine has huge significance at industrial level especially, cosmetic industry, pharmaceutical and food industry. Microbial fermentation is employed for synthesis of arginine at industrial scale [70, 71, 72, 73]. Arginine biosynthetic route of microorganisms as well as plants comprises of eight steps where first five steps lead to production of ornithine i.e. precursor for arginine [74]. Biosynthesis of arginine in case of *E.coli* follows linear route where hydrolysis of *N*-acetylornithine directly lead to production of ornithine and first enzyme *N*-acetylglutamate synthase (NAGS) is feedback inhibited by arginine and catalyzes rate determining step of route [47]. Few microorganisms like yeasts, algae and few bacteria like *P. aeruginosa* follow a different route where acetyl group from acetylornithine is recycled to glutamate and in this case the *N*-acetylglutamate kinase (NAGK) is feedback inhibited by arginine [75]. Another pathway has also been reported involving novel family of transcarbamylases for biosynthesis of arginine [76]. The *N*-acetylglutamate kinase (NAGK) may serve as arginine-inhibitable or arginine-insensitive systems in different organisms.

##### N-acetyl-l-glutamate synthase (NAGS)

**Fig. 4:**
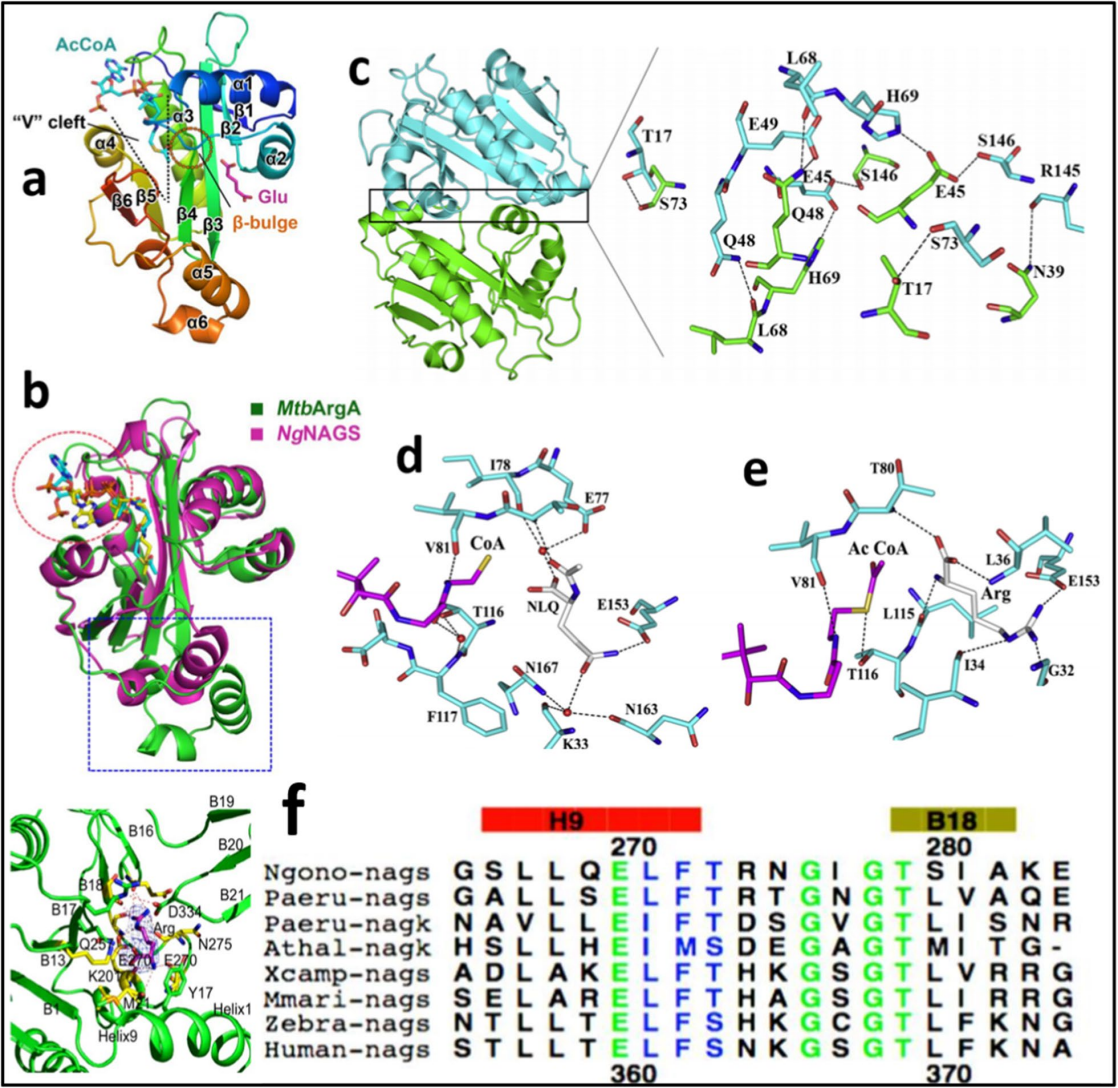
Comparison of 3D-crystal structures of NAGS from different microorganisms **a**. NAGS from *M. tuberculosis*, **b**. Superimposed structure of NAGS from *M. tuberculosis* and *N. gonorrhoeae*, **c**. Amino acids arrangement at interjunction of two monomer units, **d**. Binding of N-acetylglutamine (NLQ) and **e** arginine inside NAGS, **f**. Sequence alignment for NAGS sequences retrieved from different organisms; Reproduced from Refs. [46, 80, 81]

First step of arginine biosynthetic pathway in *E. coli* and other microorganisms following linear route is catalyzed by *N*-acetyl-l-glutamate synthase (NAGS) that is inhibited by arginine [77]. Amino acid kinase (AAK) domain of both acetylglutamate kinase (NAGK) and acetylglutamate synthase (NAGS) are similar hexameric systems formed by amino acid kinase dimers connected through N-terminal *α* helices [78, 79]. Comparison of characteristic features of 3D-crystal structures of NAGS from different microorganisms like *M. tuberculosis* and *N. gonorrhea* provided insights into conformational arrangements needed for inhibition and regulation of enzyme activity and function. Both NAGS_*M. tuberculosis*_ (PDB ID: 6ADD, 5YO2 [80] and NAGSN. Gonorrhea (PDB ID: 2R98 [81] crystal structures exists as dimer stacked together to form hexamer arrangement that is necessary for feedback inhibition by arginine. Although both NAGS comprises of distant V-cleft alongside glutamine and AcCoA binding sites still some differences were observed marked by dotted circle/box in Fig. 4a, b. The acceptor binding mode in case of NAGS_*M. tuberculosis*_ is different due to much deeper binding pocket formed by fragment (i.e. 17 residues longer than NAGS_*N. Gonorrhea*_) connecting α4 and β6. The arrangement of amino acids involved at inter-junction of two monomer units to form dimer structure are depicted in Fig. 4c [46]. Binding of L-arginine causes major changes in quaternary structure of NAGS by inducing contraction in hexamer leading to increase in width of internal cavity as revealed by differences observed in binding of arginine and N-acetylglutamine (NLQ) (Fig. 4d, e).

Sequence alignment for NAGS sequences retrieved from different organisms revealed fully conserved and partially conserved amino acids around L-arginine-binding site suggesting role of key amino acids for binding of arginine. Mutations at these sites most probably deregulate feedback inhibition caused by arginine and will aid in discovery of feedback resistant strains with improved production of L-arginine. Li et al. reported comparison of two forms of *N*-acetyl-l-glutamate synthase (NAGS) belonging to *N. gonorrhoeae* i.e. l-arginine bound T-form (inactive) and R-form (active) with bound CoA and L-glutamate [47, 81, 82]. These NAGS are comprised of single polypeptide chain having two domains i.e. N-terminal AAK (L-arginine binding site) and C-terminal NAT domain (substrate binding site).

In case of Yeast like *Saccharomyces cerevisiae*, the NAGS and NAGK play their role as complex and are targeted by feedback inhibition of arginine [81, 83, 84]. In addition, previous studies showed that bifunctional NAGS/NAGK complex reported from *Maricaulis maris* and *Xanthomonas campestris* have capability to oligomerize as tetramer where both NAT and AAK domains are similar to NAGS of *N. gonorrhoeae* but differences are observed for linker between two domains as well as their relative orientations [85]. Furthermore, it was revealed that NAGS belonging to both human and mouse are tetrameric in form just like bacterial NAGS/NAGK complex [86].

##### N-acetyl-l-glutamate kinase (NAGK)

N-acetyl-l-glutamate kinase (NAGK) also termed as argB is rate limiting enzyme of cyclic route for l-arginine biosynthetic pathway as in case of Corynebacteria where acetyl group of N-acetyl-ornithine is recycled to generate l-glutamate [72, 73, 87]. Yet, metabolic control should occur on the production of acetylglutamate, regardless of its origin. Therefore, feedback inhibition on both the synthase and the kinase is believed to be general for organisms using cyclic ornithine synthesis. [88, 89]. In contrast, NAG synthetase would appear to be a less suitable target, because in those organisms that recycle the acetyl group in the route of ornithine synthesis it only plays a purely anaplerotic role [90].

The key structural features of NAGK (3D-crystal structure; Protein data bank) from various microorganisms have been summarized in Tables 2, 3 to provide a glimpse of conformational arrangements governing feedback inhibition of arginine. The differences among arginine-sensitive and arginine-insensitive NAGK will provide clue about the discovery of new mutant structures with better production of arginine.

**Table 2:** Characteristic features of NAGK (3D-crystal structure) from various organisms

Enzyme	Structural composition	Key structural features	3D-view of structure (cartoon representation)	Reference
NAGK (Yeast)	Tetramer	Central structure is a flat tetramer formed by two dimers of AAK domains Bound arginine and NAG		[91]
NAGK (E.coli)	Homodimer	Each subunit comprised of two lobes (i.e. C-lobe binds ADP moiety of ATP and N-lobe forms inter subunit surface Arginine free		[47, 92]
NAGK (T. maritima)	Hexamer	Formed by linking three E. coli NAGK-like homodimers through the interlacing of an N-terminal mobile kinked a-helix Arginine is bound in each subunit flanking the interdimeric junction, in a site formed between the N helix and the C lobe of the subunit		[47]
NAGK (P. aurogenosa)	Hexamer (Ring-like)	Arginine free Bound NAG and ADP-Mg		[47, 91]
NAGK (C. crenatum	Swiss model hexamer (Ring-like)	Arginine binding domain comprised of Glu19, His26, Arg209 and His268 Glu19 at entrance of binding site is key amino acid		[93]

**Table 3:** Structural characteristics of various enzymes targeted by amino acid feedback inhibition

Enzyme	Feedback Inhibitor	Pathway	Structural characteristics	References
Dihydrodipicolinate synthase (DHDPS)	L-lysine	Diaminopimelic acid pathway (DAP)	i. Either as dimer or tetramer (more stable) ii. DHDPS has different level of sensitivity towards L-lysine based on its source/origin iii. DHDPS of gram-negative are lysine-sensitive and DHDPS of gram-positive are lysine-insensitive	[68, 139–149]
Homoserine *O*-acetyltransferase (HTA)	L-methionine	Aspartate biosynthetic pathway; Branched methionine biosynthetic pathway	Comprises of two domain i. core *α*/*β*-domain (comprising of eight strands; parallel *β*-sheets *β*1, *β*4–*β*10 connected to five *α*-helices on one side and one on other side ii. Helical bundle domain (composed of five *α*-helices) that form a lid over core domain iii. tunnel at interface of two domains that serve as channel for transfer of substrate to active site	[150–154]
chorismate mutase/prephenate dehydrogenase (TyrA)	Tyrosine	Aromatic amino acids biosynthesis (i.e. tyrosine)	i. Homodimer where N-terminal CM domain catalyzes conversion (Claisen rearrangement) of chorismate to prephenate that is later converted to 4-hydroxyphenylpyruvate through C-terminal PDH domain ii. Dimer in case of *H. influenza* where each monomer consists of N-terminal: NAD^+^ binding site and C-terminal *α*-helical dimerization domain iii. Tyrosine being competitive inhibitor binds directly inside active site	[116, 155, 156]
Serine acetyltransferase (SAT)	Cysteine	cysteine biosynthesis	i. Exists as monomer, trimer and hexamer ii. Monomer comprises of two domains i.e. (i). *α*-helical domain (residues 1–140), (ii). *β*-helical domain (residues 141–262) iii. Monomer structural units pack together to form trimer structure and ultimately hexamer structure iv. cysteine binding site is located at interface of two subunits	[48, 157–159]
3-phosphoglycerate dehydrogenase Phosphoserine phosphatase (PSP)	L-serine	Glycolate and phosphorylated pathway	i. Homotetrameric structure comprising of four active sites alongside four effector binding sites ii. Ligand/inhibitor binding site lies in regulatory domain region iii. PGDH_Mt_ exits in three different forms i.e. homodimer, homotetramer, homooctamer iv. Different level of sensitivity towards L-serine inhibition	[160–167]
Pyrroline-5-carboxylate synthase Glutamate kinase (GK)	Proline		i. Exists as dimer, tetramer and hexamer structure ii. Feedback inhibition of GK by proline is not affected by oligomeric structure iii. Different arrangements of subunits are observed for dimers in case of *C. jejuni* and *E.coli*	[60, 168, 169]

Arginine insensitive NAGK belonging to *E. coli* are homodimer while those arginine sensitive type of NAGK are hexamer comprising of three homodimeric units structurally similar to NAGK of *E. coli*. Arginine feedback inhibition is not necessarily attributed to hexameric form but hexamer system play major role for proper functioning and stability of NAGK and enhances arginine sensitivity [94].

### Aspartate family of amino acids

Aspartate family includes lysine, threonine and methionine that regulate their own synthesis by end product feedback inhibition. Lysine and threonine synthesis is achieved both by DAP biosynthetic pathway or AAA biosynthetic pathway and enzymes catalyzing committed step of these routes are feedback inhibited by end product amino acids. The key enzyme targeted by lysine feedback inhibition are dihydrodipicolinate synthase (DHDPS), aspartokinase (AK) and while threonine feedback inhibit Homoserine kinase (HSK). Similarly, Homoserine *O*-acetyltransferase (HTA) is feedback inhibited by methionine.

*Aspartokinase III* The aspartate kinase the first enzyme of aspartate pathway is feedback inhibited by l-lysine in *C. glutamicum*. Three forms of aspartokinase (AK I, AK II and AK III) have been reported in different bacterial species. In case of *B. subtilis* and *E. coli*, the aspartokinase II and aspartokinase III are feedback inhibited by l-lysine and l-threonine [95, 96].

The regulation of lysine biosynthesis varies among organisms. Only aspartokinase is regulated for the biosynthesis of lysine in *C. glutamicum*. It is inhibited by l-lysine plus l-threonine; either l-lysine or l-threonine alone does not inhibit aspartokinase. In *E. coli*, many enzymes of lysine biosynthesis are regulated by the end-products of the pathway. There are three isozymes of aspartokinase in *E. coli*: aspartokinase I is repressed by l-threonine plus l-isoleucine and inhibited by l-threonine, aspartokinase II is repressed by l-methionine, and aspartokinase III is repressed and inhibited by l-lysine. Aspartokinase I and II are bifunctional enzymes and show both aspartokinase and homoserine dehydrogenase activities [97–99].

**Fig. 5:**
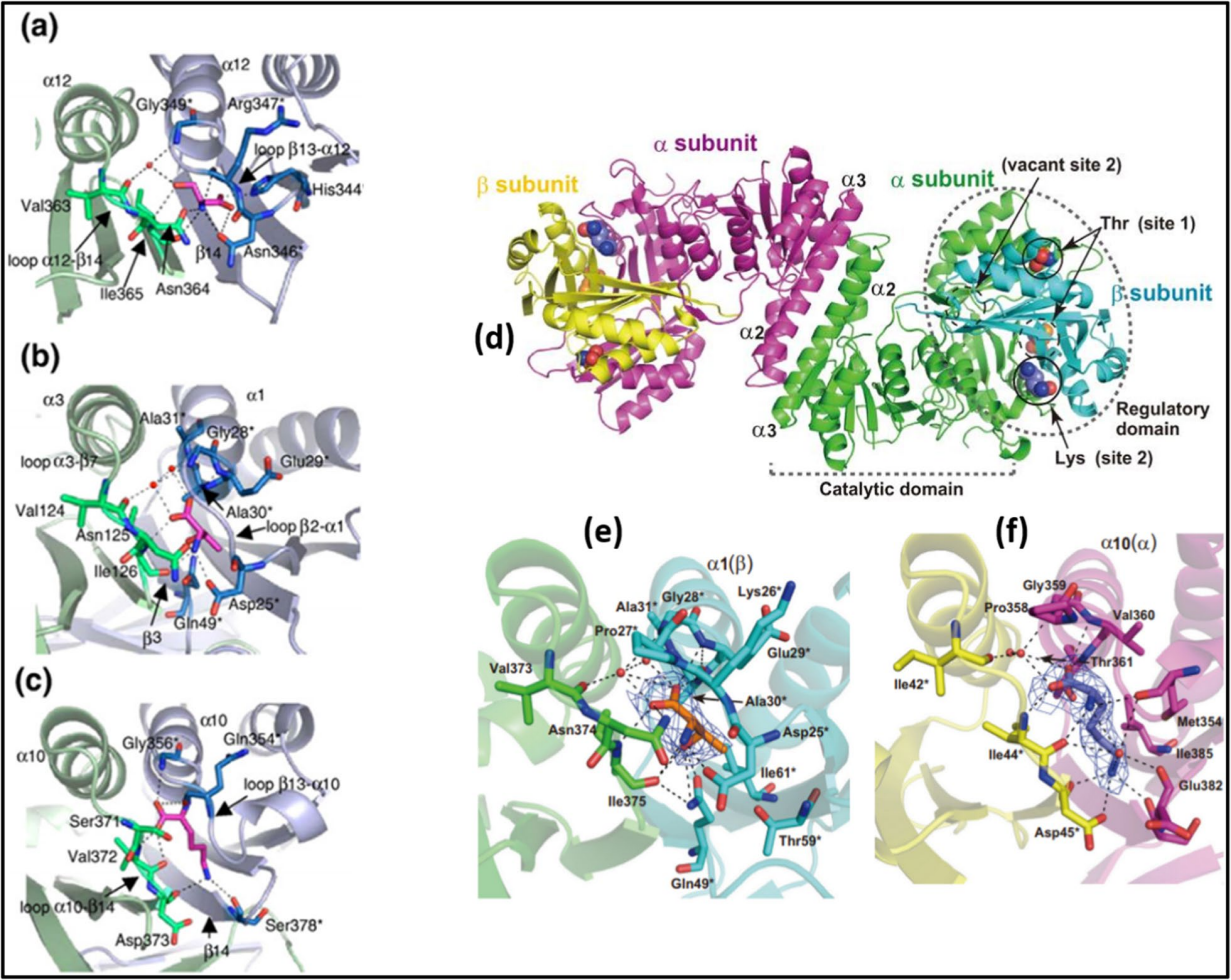
Comparison of ACT domains of **a**. Ser-binding site of 3PGDH, **b**. Thr-binding site of CgAKβ, **c**. Lys-binding site of AtAKI **d**. Overall structure of α_2_β_2_-type aspartokinase (AK) from *C. glutamicum*, **e**. Threonine-binding site of AK of *C. glutamicum*, **f**. Lysine-binding site of AK of *C. glutamicum*; Reproduced from Refs. [100, 101]

Comparing crystal structure (retrieved from protein data bank) of aspartokinase from different microorganisms namely *C. glutamicum* and *E.coli* [54, 100, 101] revealed presence of ACT domains in monomer, dimer or tetramer structures. This ACT domain is characteristic feature of various kinases, 3PGDH, AK-I and AK-III where ligand binds i.e. serine, threonine and lysine binds similar region as depicted in Fig. 5.

Comparison of AK_*C. glutamicum*_ and AK_*E.coli*_ identified presence of two state conformation (T-state and R-state) in case of *E. coli* where each monomer unit is comprised of two ACT domain at C-terminus while each monomer unit of tetramer structure of *C. glutamicum* has single ACT domain [101, 102]. The AK from *C. glutamicum* is feedback inhibited by both lysine and threonine in a concerted way while AK of *T. thermophilus* is feedback inhibited by threonine only [103–105]. Ayako et al., reported crystal structure of AK from *C. glutamicum* as: inhibitory complex bound to lysine and threonine, (ii). Active complex bound to threonine. Comparison of these two forms of AK revealed that inhibitory form is stabilized by conformational changes caused by binding of inhibitors. Structure of AK is heterotetramer comprised of two *α* subunits and two *β* subunits where dimerization of homo-oligomeric AK involves interaction among regulatory domains. The crystal structure with bound lysine and threonine exists as in active T-form [101]. AK structure bound to both lysine and threonine follow a concerted mechanism for inhibition involving binding of threonine first following lysine binding lead to closed conformation Fig. 5.

### Enzyme targeted by feedback inhibition of histidine

#### ATP-phosphoribosyl transferase (ATP-PRT)

Histidine biosynthetic pathway found in bacteria, plants and fungi comprises of ten steps and has been considered as well recognized target for discovery of various antimicrobial drugs. The enzyme ATP-phosphoribosyl transferase (ATP-PRT) catalyzing first committed step of pathway is feedback inhibited by end product histidine. The reaction catalyzed by ATP-PRT involves synthesis of N′-5′-phosphoribosyl-ATP (PR-ATP) by condensation of ATP with 5-phosphoribosyl-a-1-pyrophosphate (PRPP) [106]. The enzyme ATP-PRT play major role to regulate microbial growth as histidine biosynthetic pathway is connected with various other metabolic pathway [51, 107]. Histidine allosterically inhibit ATP-PRT enzyme at site other than active pocket termed as allosteric site while AMP, GTP and ADP serve as competitive inhibitors [50].

Crystal structural analysis of tertiary and quaternary ATP-PRT revealed two different forms. The most widely found homo-hexameric form, encountered in bacteria, fungi, and plants, while second form i.e. the hetero-octameric type, limited to few bacterial strains. ATP-PRT belonging to *M. tuberculosis* is homo-hexameric class, also found in *S. enterica* and *E. coli* [51, 108]. Crystallographic data revealed that L-histidine binding converts active dimer of MtATP-PRT into an inactive hexamer [50]. Furthermore, the L-histidine-bound hexameric form causes major conformational changes, having twist of domain III compared to domain I and II. Crystallographic studies also revealed that L-histidine binds at a site approximately 30 Å away from the active site, suggesting allosteric nature of inhibition. Finally, it was observed that L-histidine interacts with allosteric site involving carboxyl group of Asp218, the hydroxyl moiety of Thr238, and the backbone amide oxygen of Ala273 [50]. The mycobacterial ATP-PRT enzyme comprises of ten helices and 15 *β*-strands arranged as three domains. Domain I contains a central *β*-sheet consisting of four parallel *β*-strands and two anti-parallel strands while domain II also has four parallel *β*-strands and one anti-parallel *β*-strand surrounded by two α-helices on each side. Catalytic site (substrate binding site) of ATP-PRT lies at intersection of domain I and II involving mostly interaction with amino acids of domain II while allosteric inhibitor histidine binds in domain III. The catalytic domain of ATP-PRT of both mycobacteria and glutamine binding *E. coli* are similar to each other [50, 109].

**Fig. 6:**
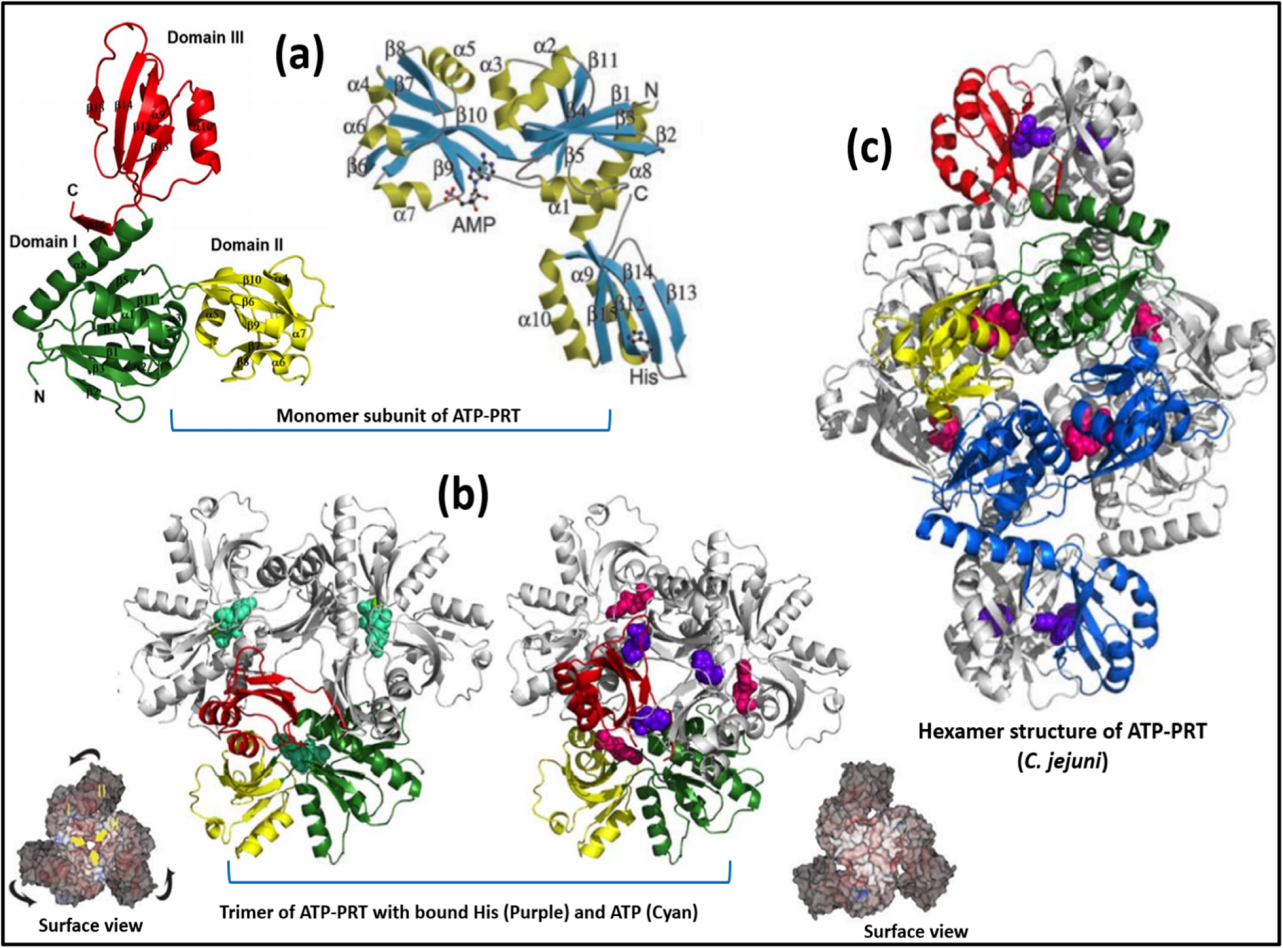
**a**. Cartoon representation of the ATP-PRTase monomer (*M. tb* and *C. jejuni*) where ligand AMP and His are shown in ball-and-stick, **b**. Cartoon representation of ATP-PRT trimer structure ATP (open domain III domain) and His bound (More close domain III domain), **c**. Hexamer structure of ATP-PRT (*C. jejuni*) with bound His, and AMP; Reproduced from Ref. [49] and [50]

The ATP-PRT enzyme of proteobacteria has shorter form without domain III where HisG forms complex with HisZ. Crystal structure of both apo and AMP:His complex is in hexamer form comprised of trimer of dimers where histidine bound hexameric complex is more compact than apo form as shown in Fig. 6. In case of dimer, the interactions mainly involve domain I and II of catalytic domain while hexamer interface involve histidine binding domain (domain III). Histidine binding stabilizes hexamer form (inactive) and also influences catalytic site for substrate binding due to steric hindrance [50].

Histidine binding alongside AMP causes conformational changes in ATP-PRT structure where active site of enzyme is closed and amino acid residues involved in binding of PRPP also disrupt that hindered catalytic activity [50].

### Aromatic amino acids family of amino acids

#### Enzyme targeted by feedback inhibition of tryptophan

Aromatic amino acids namely tryptophan, phenylalanine and tyrosine follow Shikimate pathway for their biosynthesis. Despite their role as building block for protein, they also serve as precursor for various pharmaceutical products. For instance, Tryptophan serve as precursor for various metabolites with significant pharmacological importance especially, vinblastine and vincristine two well-known anticancer drugs while Tyrosine is precursor for neurotransmitter dopamine, p-hydroxystyrene and p-hydroxycinnamic acid, needed for manufacturing of novel materials, pharmaceuticals and nutraceuticals [110–115]. Chorismate is a branch point biosynthesis of phenylalanine, tyrosine and tryptophan alongside other metabolites. The shikimic acid pathway of bacteria is regulated and homeostasis is maintained through end product feedback inhibition for enzyme of branch point [116]. The Anthranilate synthase (AS) catalyzes first committed step of pathway and is feedback inhibited by Tryptophan. On the other hand, the l-phenylalanine and l-tyrosine are formed from chorismic acid via prephenate, which undergoes either decarboxylation/dehydration or decarboxylation/dehydrogenation, followed by a transamination to generate given amino acids. The enzymes namely prephenate dehydrogenase and prephenate dehydratase catalyzes first step of tyrosine pathway and phenylalanine pathway, respectively and are feedback inhibited by their end product.

##### Anthranilate synthase (AS)

Tryptophan belong to aromatic amino acids family and its biosynthesis proceeds via a common pathway to chorismate [117]. The first step of tryptophan biosynthetic pathway is catalyzed by anthranilate synthase (AS; EC 4.1.3.27) that is feedback inhibited by final product i.e. tryptophan. Anthranilate synthase comprises of two polypeptide chains that form a complex TrpE_2_:TrpG_2_ and catalyzes the first reaction branching from the shikimate pathway toward the biosynthesis of tryptophan occurring in bacteria, fungi and plants [118]. AS is composed of two non-identical subunits where TrpE subunit binds chorismate (CA) and is the site of formation of anthranilate from CA and NH_3_ while TrpG belongs to glutamine amidotransferase family [119]. The reactions catalyzed by TrpE and TrpG involves: (i) Glutamine-dependent AS reaction requires both subunits while (ii) Ammonia-dependent AS reaction requires either *α*-subunit or both subunits [120]. Glen et al. reported the crystal structure of anthranilate synthase (AS) from *Serratia marcescens* in the presence of its substrates and inhibitor l-tryptophan. The trpE of *S. marcescens* comprises of domain I (composed of 1–57, 178–308, 468–520 amino acid residues) and domain II (composed of antiparallel *β*-sheet from 9 to 12 strands (*β*4, *β*4a, *β*4b) alongside two *α*-helices (*α*1a,*α*1b). The junction of two domains contains various amino acids causing tryptophan feedback inhibition while another neighboring crevice attached to trpG contains amino acid residues involved in substrate binding [63].

**Fig. 7:**
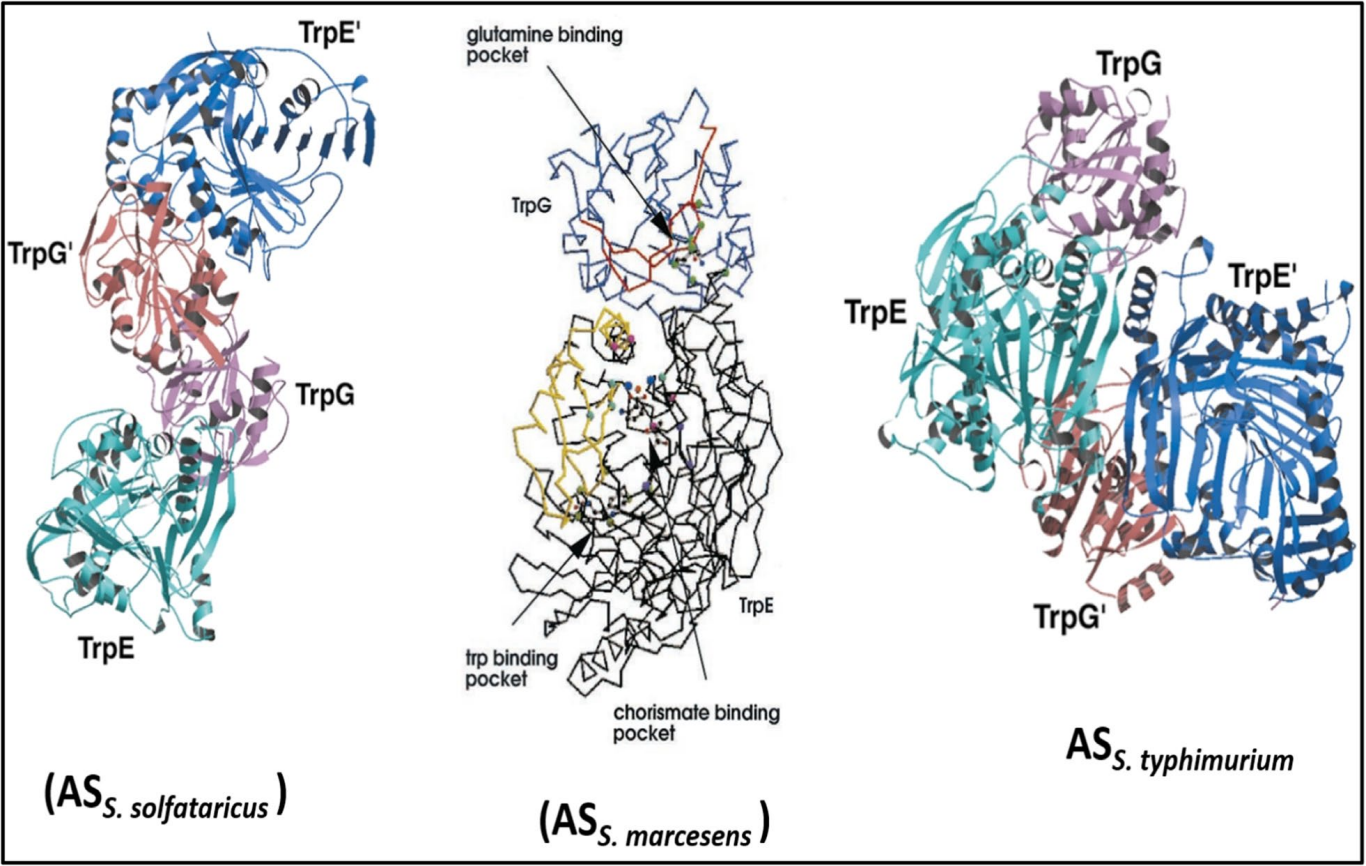
Comparison of Anthranilate synthase structure from *S. solfataricus*, *S. marcesens* and *S. typhimurium*; Reproduced from Ref. [63]

Comparison of crystal structures of AS from various organisms revealed different arrangement of TrpG and TrpE in heterotetrameric complex. The structural composition of AS from *S. marcesens* is comparable to AS from *S. solfataricus* while AS of _*S. typhimurium*_ represents different structure as depicted in Fig. 7.

Structural characteristics of TrpG and TrpE from *S. marcescens* is comparable to *S. solfataricus* where Gly328, Thr329, His398 and Gly485 from chorismate binding domain play vital role in binding of chorismate while Ser40, Pro291, Met293, Val453, Tyr455 are involved in binding of tryptophan. Similarly, glutamine, pyruvate and benzoate binding site are also shown as different regions in Fig. 6a, b. Anthranilate synthesis need both sub units (*α*- and *β*) of anthranilate synthase involving reaction of chorismate with glutamine while only *α*-subunit is needed for reaction of chorismate and ammonia (at high concentrations of ammonium) [121, 122].

The second enzyme of tryptophan biosynthetic pathway anthranilate phosphoribosyl transferase (AnPRT) is also feedback inhibited by L-tryptophan. The AnPRT (TrpD) catalyzes reaction of PRPP and anthranilate to N-(5′-phosphoribosyl)-anthranilate (PRA) and PPi. Crystal structure revealed AnPRT as homodimer having N-terminal domain comprising of six *α*-helices and C-terminal domain formed by eight *α*-helices surrounding seven stranded *β*-sheet [123]. The active site is present at interface of C- and N-terminal domains as revealed by crystal structure of *S. solfataricus*, *P. carotovorum*, *T. thermophiles*, *X. campestris*, *M. tuberculosis* [124–128].

The enzyme anthranilate phosphoribosyl transferase (AnPRT) belonging to enteric bacteria exists as complex with anthranilate synthase (AS). Bifunctional TrpD: TrpE complex is responsible for the first step in tryptophan biosynthesis i.e. the transfer of an amino group from glutamine to chorismate and the formation of anthranilate. The Feedback inhibition of anthranilate phosphoribosyl transferase (AnPRT) is observed in its complex form with AS and sensitivity to tryptophan feedback inhibition is lost once complex is distorted [129].

### Pyruvate family of amino acids

#### Enzyme targeted by feedback inhibition of leucine

The branched chain amino acids follow pyruvate pathway for their synthesis and use pyruvate or 2-ketobutyrate as precursor formed through acetohydroxy acid synthase (AHAS). The pyruvate pathway branches at isopropylmalate (IPM) where 2-ketoisovalerate and acetyl CoA is converted to *α*-isopropylmalate catalyzed by *α*-isopropylmalate synthase (*α*-IPMS) regulated by leucine feedback inhibition [130]. The IPM pathway has been reported as key route for synthesis of leucine for bacteria, fungi and plants [131–133].

##### *α*-isopropylmalate synthase (*α*-IPMS)

**Fig. 8:**
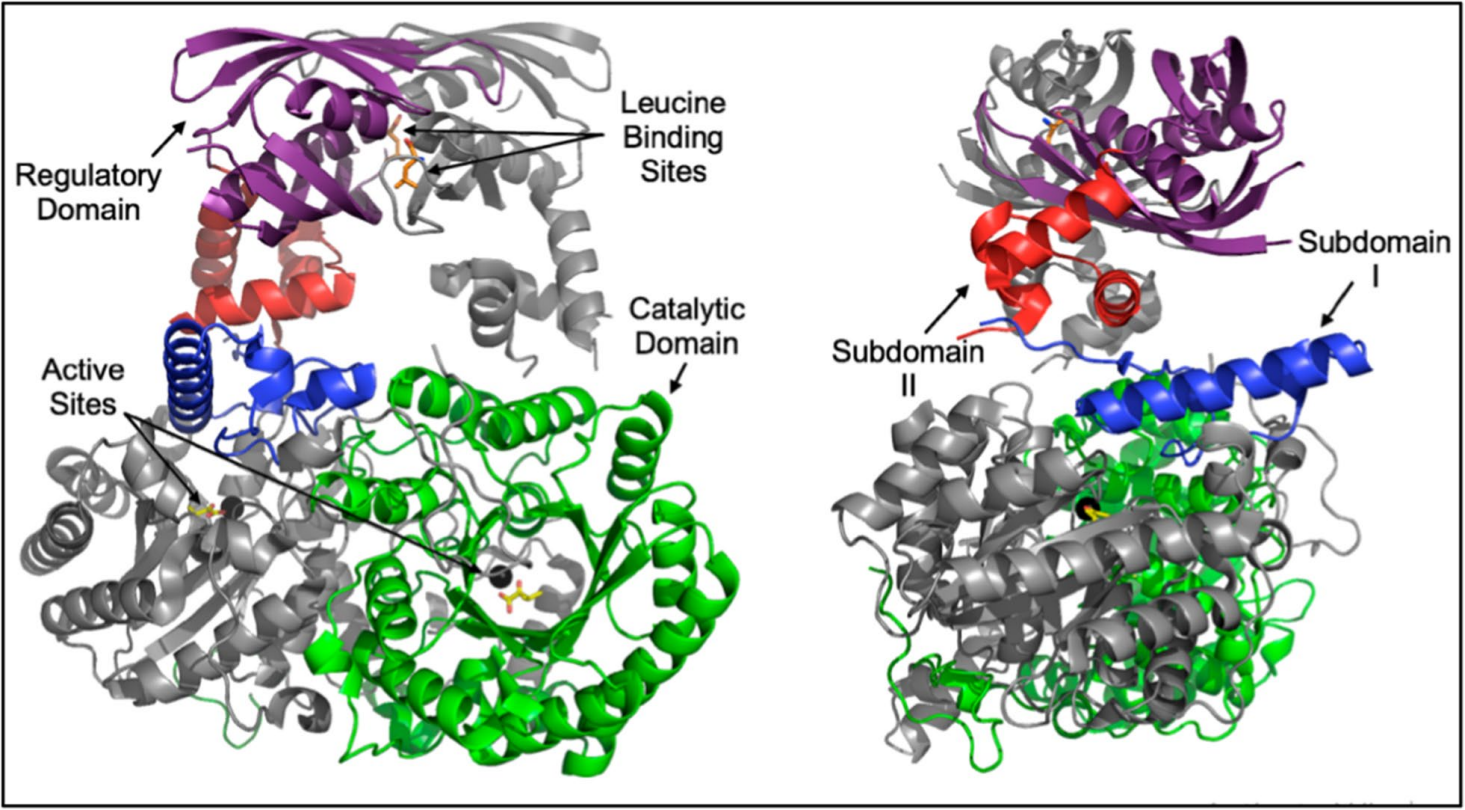
Crystal structure of *α*-IPMS from *M. tuberculosis*; Reproduced from Ref. [136]

The *α*-isopropylmalate synthase catalyzing first committed step of leucine biosynthetic pathway branched from pyruvate route is feedback inhibited by end product leucine. The reaction catalyzed by IPMS involves Claisen condensation of *α*-ketoisovalerate and AcCoA to produce *α*-isopropylmalate [134]. Luiz et al., reported crystal structure of *α*-IPMS from *M. tuberculosis*, a monomeric structure comprising of two domains i.e. N-terminal (catalytic site) and C-terminal (regulatory site) connected through two subdomains depicted in Fig. 8. The catalytic domain exits as (*α*/*β*)8 TIM barrel where linker domain I comprises of *α*10 and two short *β*-strands, while subdomain II composed of *α*11-*α*13. Similarly, regulatory domain located at C-terminal is comprised of two *βββα* units. They also identified that C-terminal domain markedly effect activity of enzyme [135, 136].

Structural analysis of *α*-IPMS revealed binding of Zn^2+^ and *α*-ketoisovalerate into active site while leucine exhibits reversible feedback inhibition by binding inside regulatory domain. Feedback inhibition of *α*-IPMS by leucine follow time dependent slow onset inhibition where complex formed by leucine binding inside regulatory domain isomerizes to tightly bound complex. The linker domain facilitate in transmission of inhibitory signals towards catalytic site [134]. Cavalieri et al., reported L-leucine insensitive *α*-IPMS from *S. cerevisiae* mutations at leucine binding site [137] while another mutant structure reported for *α*-IPMS_Mtb_Tyr410Phe mutant also showed insensitivity to leucine binding [138].

## Deregulation of amino acids feedback inhibition

### Applicability of basic principles for deregulation of histidine feedback inhibition (Case study I)

It is a generalized fact that structural characteristics of given enzyme system has marked effect on deregulation of feedback inhibition of amino acids. Enzymes existing as monomer, dimer or oligomer complexes comprised of different domains, chains, and sites. Mutations at any of these sites negatively effects binding of inhibitors and ultimately inhibition tendency alongside catalytic activity of that enzyme complex is disrupted. Here, we discussed applicability of various mechanisms and strategies employed to accomplish deregulation of feedback inhibition for histidine biosynthetic pathway (i.e. taken as example).

Histidine has huge significance as per its role in various pharmaceutical products and also serve as precursor for production of various bioactive compounds. Owing to its role in various industries like pharmaceutical and cosmetic industry, the aim of biotechnologists is to enhance its production at industrial scale by implementing various available techniques and approaches. Bioinformatician, as well as experimentalists are contributing their efforts to accomplish deregulation of enzyme′s feedback inhibition. Commonly used strategies are (1). Reducing interaction and binding of feedback inhibitor inside pocket, (2). Blocking entry of inhibitor into binding domain, (3). Mutations at other parts/sites of enzyme to eliminate metastability of protein due to inhibitor binding, (4). Effect of mutations at interface of dimer or multimer enzyme, (5). Competitive feedback inhibition by end product (i.e. amino acid). The structural characteristics of ATP-phosphoribosyl transferase (ATP-PRT) enzyme (feedback inhibited by end product histidine) revealed two forms i.e. homo-hexamer or hetero-octamer. Detailed discussion over use of various techniques and strategies for deregulation of feedback inhibition in HisG are provided in next section.

*Reducing binding of feedback inhibitor inside pocket/regulatory domain* Allosteric inhibitor binding sites have been previously engineered by various research groups to accomplish reduced binding of inhibitor inside pocket. Reducing interaction tendency of inhibitor by mutations of key amino acids of binding domain is of paramount importance for deregulation of feedback inhibition. Cho et al., reported crystal structure of ATP-Phosphoribosyl transferase (HisG) and identified key amino acids involved in binding of allosteric inhibitor histidine [50]. Zhang et al. employed this approach of reducing binding interaction with pocket and reported site directed mutagenesis of key amino acids (Asn215, Leu231, Thr235, and Ala270) involved in feedback inhibition identified through sequence identity comparison of HisG from *M. tuberculosis* and *C. glutamicum*. The N215K/L231F/T235A mutant of HisG resulted in higher yield of histidine [170]. Similarly, G233H/T235Q mutants of Gly233 amino acid residue reported by Schendzielorz and co-workers enhanced production of histidine [171]. Similar approach was utilized against DHDPS from *C. glutamicum* where four amino acids of regulatory domain directly interacting with l-lysine were mutated by Feng et al. [89]. They reported total deregulation of feedback inhibition for His56Lys and Glu84Thr suggesting essentiality of these two amino acids for l-lysine binding and inhibition. As mentioned earlier, the histidine binding into allosteric site (Domain III) of ATP-PRT results in more compact and inactive form of enzyme. Identifying key amino acids involved in binding of L-histidine like Asp218, Thr238, and Ala273 [146] and sequence identity comparison among ATP-PRT of different sources will facilitate in achieving mutations at these sites to accomplish deregulation. Computational as well as experimental mutagenesis approaches can be employed. Similarly, comparison of feedback sensitive and feedback insensitive forms of ATP-PRT enzyme belonging to different sources through sequence identity calculation also facilitate to discover new enzyme variants of interest. For instance, Yoshimi et al., reported three mutants of aspartokinase III i.e. Thr344Met, Ser345Leu, and Gly323Asp with considerable lysine-insensitivity [172] while Met318Ile and Thr352Ile of *E. coli* AKIII (lysine-insensitive AKIII) were reported by Falco et al. [173].

**Fig. 9:**
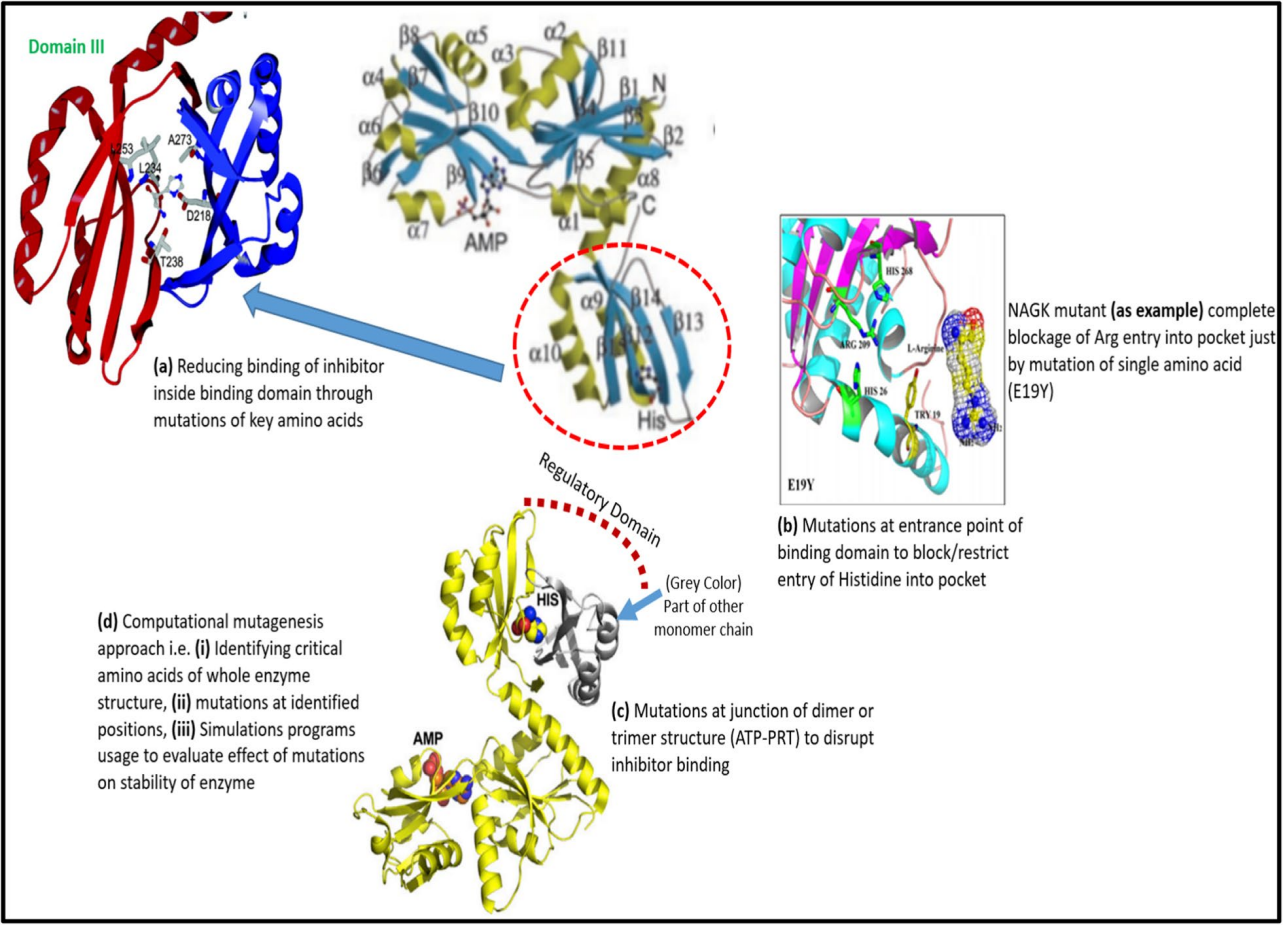
Strategies for deregulation of feedback inhibition in HisG

Blocking entry of inhibitor into binding domain Modifying the key amino acid residues positioned at the entrance of feedback inhibitor binding site facilitate to block entry of inhibitor Fig. 9a, b. Steric hindrance, change in hydrophobicity or hydrophilicity and charge modification are key contributor to achieve deregulation of feedback inhibition in this case. This could be an effective strategy to achieve deregulation of ATP-PRT enzyme as previously reported for deregulation in feedback inhibition for NAGK from *C. crenatum* by mutations at Glu19 position located at entrance of arginine binding site. Where the feedback inhibition of l-arginine was most deregulated in the Glu19Tyr, Glu19Trp and Glu19Phe mutants [93]. Now, in case of ATP-PRT enzyme histidine bound structure exists as trimer or hexamer form where histidine binding site is located at junction of two monomeric units and blocking entry of histidine in this site can be achieved by mutations of amino acids at entrance point.

*Eliminate metastability of protein due to inhibitor binding* As histidine binding introduces compactness in trimeric, or multimeric structure of ATP-PRT enzyme. Mutations or engineering of sites other than feedback inhibitor binding site may cause conformational changes alongside disruption of enzyme stability that ultimately destroy catalytic activity of enzyme. Owing to effect of histidine binding with key amino acids of binding domain, the modifications on other sites or positions of enzyme cause conformational changes and allosterically effect catalytic activity as well as inhibitor binding tendency of ATP-PRT. Plenty of approaches i.e. both experimental and computational are in common practice to introduce mutations in enzyme structure for discovery of new enzyme variants. This particular approach has been successfully employed by various research groups for aspartokinase III to identify enzyme mutant structures with deregulated inhibition for instance Gln298Gly and Ala279Thr (using point mutation approach) [174–176].

*Effect of mutations at interface of dimer or multimer enzyme* Histidine need ATP-PRT enzyme in trimer or hexamer form to show its feedback inhibition as Histidine binding domain lie at junction of two monomeric units. Mutations at interface of dimer structure may disrupt inter-domain communication and transfer of signals leading to loss of catalytic activity and feedback inhibition as shown in Fig. 9c, d. Most of enzymes targeted by feedback inhibitor exists as dimer, tetramer or hexamer structure and mutations at junction of these monomeric units may affect feedback inhibition, on the other hand catalytic activity of enzyme is also effected and coping this situation is of paramount importance. This approach have been successfully employed to achieve deregulation of *α*-IPMS (mutant Y410F) with complete loss of sensitivity towards feedback inhibition by leucine [138].

As histidine has its own binding domain/regulatory domain and its interactions with pocket affects catalytic activity through allosteric effect. On the other hand, few amino acids like L-threonine showed feedback inhibition as competitive inhibitor and disrupting their binding inside active pocket will ultimately destroy the catalytic activity. Multiple strategies could be employed to retain wild-type activity while decreasing affinity for the competitive inhibitor. Like engineer new interactions that support substrate binding but weaken inhibitor binding, effecting hydrophobicity, creating steric bulk or decreasing strength of van dar Waals interactions.

Plenty of mutagenesis approaches including both experimental and computational techniques are in common practice to achieve deregulation in targets of interest. Commonly used in vitro mutagenesis techniques are Directed evolution technique [177, 178], Site directed mutagenesis (SDM) [179], Site saturation mutagenesis (SSM) [180, 181], Random mutagenesis [182], Chemical mutagenesis, Error-prone PCR (ep-PCR) [183, 184], Rolling circle amplification-PCR (RCA-PCR) [185–188]. The summary of mutant structures of enzymes with deregulated feedback inhibition by amino acids have been provided in Table 4.

**Table 4:** Mutations reported for deregulation of feedback inhibition by amino acids against enzyme targets

Enzyme	Mutants	Deregulation of Feedback Inhibition	Reference
NAGK (*C. glutamicum*)	H271N and E284D E19R, H26E, R209A, H268N, G287D	Markedly enhanced Arginine production Moderate effect on inhibition tendency of Arginine	[189]
NAGK (*C. crenatum*)	E19F, E19L, E19A E19G, E19R	Restrict binding of arginine Enhanced arginine tendency	[190]
HisG (*C. glutamicum*)	N215K/L231F/T235A G233H/T235Q S143F	Increased Histidine production Strongly deregulated Histidine Feedback inhibition	[170, 171]
DHDPS (*C. glutamicum*)	H56K, E84T A49P, A49W, L51T	Total deregulation of Histidine feedback inhibition Slight deregulation of Histidine Feedback inhibition	[97]
DHDPS (*E. coli*)	R138H, R138A	–	[68]
DAPDC	Q381A, R385A	Negative effect on catalytic activity	[191]
Aspartokinase III (*E. coli*)	T352I, S300T S369F, E164K T344M, S345L, G323D	Total deregulation of feedback inhibition No effect of feedback inhibition Considerable lysine insensitivity	[98, 172, 192]
Aspartokinase (*C. glutamicum*)	Q298G, I272E, N372A, I375P Glu382Ala T361A, S381F, A279T	Complete deregulation of Lysine feedback inhibition Moderate deregulation of Lysine feedback inhibition No effect on deregulation Highest resistance towards feedback inhibition of Lysine	[100, 174]
AKI-HDI (*S. marcescens*)	S352F	Highest resistance towards feedback inhibition	[193]
HSK (*C. glutamicum*)	A20G	Significantly reduces threonine feedback inhibition	[194]
HSK (*E. coli*)	H202L, R234L, R234C, R234H, H139L	Marked effect on catalytic activity	[195]
SAT (*E. histolytica*)	H208S	Marked decrease in cysteine sensitivity	[196]
SAT (*E. coli*)	M256I, M201V, E166G	Significant insensitivity towards Cysteine inhibition	[197, 198]
α-IPMS (*M. tuberculosis*)	R80A, R80K, K79A, N83A Y410F, E218A	Destroyed enzymatic activity Slow onset of feedback inhibition Complete loss of leucine sensitivity	[138, 199]
Chorismate mutase (*S. cerevisiae*)	E246Q N194D	Profoundly affect catalytic activity No effect on catalytic and regulatory properties	[200, 201]
Chorismate mutase (*E. coli*)	A354V and F357L	Tyrosine resistant	[202]

No significant studies have been reported for mutational studies over Anthranilate synthase (AS), Anthranilate phophoribosyl transferase (AS-PRT), Pyrroline carboxylase reductase, Glutamate 5-kinase (GK), and Homoserine-O-acetyltransferase (HSAT) enzyme targets regulated by feedback inhibition of their own end product amino acids.

In addition, the development of bioinformatics algorithms enabled computational approaches to provide more precise guidance for enzyme engineering and make it more efficient and less laborious. The success of rational design depends on in-depth knowledge about sequence and structure features of target proteins. Various computational mutagenesis approaches have been reported to get in depth sights of protein functions, structure, stability and thermodynamic characteristics (Table 5). The folding and interaction among various amino acid residues of globular proteins depend on proteins sequences while mutating these amino acids will disrupt these folding patterns hence different protein conformations are obtained with varied thermodynamic characteristics. For instance, computational saturation mutagenesis (CoSM) uses molecular dynamic equilibration, sidechain flips and energy minimization to improve side conformations in mutants enable prediction of stability changes with better accuracy and correlation with the experimentally deciphered stability changes. Similarly, elastic network contact model (ENCoM) based on normal mode analysis (NMA) rely on amino-acid’s nature and help to calculate vibrational entropy changes upon mutations [203, 204]. Unfolding mutation screen (UMS) another well-known computational mutagenesis technique employed to evaluate effect of given mutations on structure and functions of protein using unfolding propensity and display of data as interactive heat maps. The UMS approach is advantageous over previously used techniques as it does not need prior knowledge of protein structure and function [205–207].

**Table 5:** Various web-servers used for computational mutagenesis

WebServer	Abbreviation	Function/usage	Refs.
Protein variation effect analyzer	PROVEAN	To predict impact of amino acid substitution on the biological function of a protein	[208]
PolyPhen-2	–	For prediction of effect of amino acid substitution on the structure and functions of a protein	[209]
Swiss-model expert protein analysis system	ExPASy	A fully automated protein structure modeling server, accessible via the ExPASy web page	[210]
Screening for non-acceptable polymorphisms program	SNAP2	To incorporate evolutionary information, predict aspects of protein structure, and other relevant information in order to make predictions regarding the functionality of mutated proteins	[211]
KStable	–	It is sequence-based, computationally rapid, and includes temperature and pH values to predict changes in the thermostability of a protein upon the introduction of a mutation at a single site	[212]
Evolutionary, amino acid, and structural encodings with multiple models	EASE-MM	Sequence-based prediction of mutation induced stability changes with feature-based multiple models. This method is applicable to single-domain monomeric proteins and can predict ΔΔ*G*_*u*_ with a protein sequence and mutation as the only inputs	[213]
Allosteric signaling and mutation analysis	AlloSigMA	The AlloSigMA server is a tool for estimating the allosteric free energies acting on a single residue as a result of either ligand binding, mutations, or both combined	[214]
FoldX	–	FoldX is an empirical force field that was developed for the rapid evaluation of the effect of mutations on the stability, folding and dynamics of proteins and nucleic acids	[215]
Allosteric mutation analysis and polymorphism of signaling database	AlloMAPS	Evaluating the modulatory effects of perturbations on the allosteric regulation, and allowing estimation of allosteric effects of non-native allosteric sites and mutations	[216]
Single amino acid mutation change of binding energy	SAAMBE 3D	Machine learning algorithm to predict the effects of single amino acid mutation on PPIs	[217]

Literature revealed that none of above mentioned in silico mutagenesis techniques have been employed for mutations prediction to deregulate feedback inhibition of enzymes. Using combination of various in silico approaches to design new enzyme variants with deregulated feedback inhibition is of worth importance.

## Future perspectives

Current review emphasized need of new mutagenesis techniques to accomplish more efficient enzyme variants aimed for increased production of amino acids. The detailed description of enzyme structures here affirmed that (a). Engineering of regulatory domain is key contributor to accomplish deregulation of feedback inhibition, (b). Blocking entry of inhibitor into binding domain by modifying the key amino acid residues positioned at the entrance of feedback inhibitor binding site, steric hindrance and charge modification is of paramount importance for deregulation, (c). In case of dimer, tetramer or hexameric structures, mutations at interface or junction of two monomeric units may disrupt signal transfer and ultimately destroy enzyme activity.

Biotechnologists, microbiologists and other research groups need to develop new strategies and approaches with better efficacy. Rational mutagenesis technique like site directed mutagenesis (SDM) and site saturation mutagenesis (SSM) have many advantages over random mutagenesis approach. Amalgamation of in vitro and in silico would be more effective approach and focus should be on more reliable and efficient software designs.

## Concluding remarks

Detailed literature search over amino acid biosynthetic pathways, structural details of target enzymes feedback inhibited by amino acid, mutagenesis approaches (both in vitro and in silico) used to incorporate structural and conformational changes to deregulate their inhibition tendency have been summarized. The structure, design and mechanism of product feedback inhibition for all enzyme targets have been discussed in detail to provide an insight into structural and functional basis of amino acid feedback inhibition. Bacterial amino acids biosynthetic pathways are interconnected as different amino acids may be produced using same common precursor as starting point thus has been categorized into various groups. Majority of amino acid’s biosynthetic pathways (16 out of 20 amino acids) are regulated by allosteric feedback inhibition. Structural analysis of enzyme targets that are feedback inhibited by their own end product revealed that they exists mostly as dimer, tetramer or hexamer structure comprising of multiple subunits having more than one binding and allosteric sites. Although some enzyme targets also exist as monomer structures but allosteric inhibition is mostly observed in dimer or oligomeric systems. Traditional methods used for discovery of new bacterial strains like random mutagenesis have been replaced by rational mutagenesis approach and plenty of new computer assisted methods are in use now. Identifying key sites and domains in given enzyme structure keeping in view their role in feedback inhibition prior to mutations is of worth importance that will facilitate new enzyme discovery efficiently. Current review will provide guidelines for designing of better feedback resistant enzymes and will facilitate biotechnologists for discovery of novel enzyme variants for increased production of amino acids at industrial scale.

## Data Availability

The materials and datasets supporting the review are provided within the article.

## Abbreviations

TCA: Tricarboxylic acid
PRPP: Phosphoribosyl pyrophosphate
SDM: Site directed mutagenesis
SSM: Site saturation mutagenesis
AAA: Aromatic amino acids
NAGK: *N*-Acetyl-l-glutamate kinase
NAGS: *N*-Acetylglutamate synthase
AAK: Amino acid kinase
ATP-PRT: ATP-phosphoribosyl transferase
DAP: Diaminopimelic acid pathway
AK: Aspartokinase
DHDPS: Dihydrodipicolinate synthase
AS: Anthranilate synthase
CA: Chorismate
AnPRT: Anthranilate phosphoribosyl transferase
HSK: Homoserine kinase
SAT: Serine acetyltransferase
OASS: O-acetylserine sulfhydrylase
PSP: Phosphoserine phosphatase
GK: Glutamate kinase
ep-PCR: Error-prone PCR
RCA-PCR: Rolling circle amplification-PCR
NMA: Normal mode analysis
UMS: Unfolding mutation screen
CMP: Comprehensive mutational profile
